# The BRAIN test: a keyboard-tapping test to assess disability and clinical features of multiple sclerosis

**DOI:** 10.1007/s00415-017-8690-x

**Published:** 2017-12-04

**Authors:** Samuel Shribman, Hasan Hasan, Shahrzad Hadavi, Gavin Giovannoni, Alastair J. Noyce

**Affiliations:** 1 Department of Neurology, St. George’s Hospital, London, UK; 2 Reta Lila Weston Institute of Neurological Studies, UCL Institute of Neurology, London, UK; 3 Department of Neurophysiology, Kings College Hospital, London, UK; 4 Blizard Institute, Barts and the London School of Medicine and Dentistry, Queen Mary University of London, London, UK; 5 Preventive Neurology Unit, Wolfson Institute of Preventive Medicine, Barts and the London School of Medicine and Dentistry, Queen Mary University of London, Charterhouse Square, London, UK

**Keywords:** Multiple sclerosis, Remote monitoring, Keyboard, Online, Pyramidal dysfunction, Cerebellar dysfunction

## Abstract

**Background:**

The BRadykinesia Akinesia INcordination (BRAIN) test is an online keyboard-tapping test previously validated as a sensitive tool for detecting signs of Parkinson’s disease.

**Objectives:**

To determine whether the BRAIN test can measure disability in MS and identify the presence of pyramidal or cerebellar dysfunction.

**Methods:**

Kinesia scores (KS, number of key taps in 30 s), akinesia times (AT, mean dwell time on each key) and incoordination scores (IS, variance of travelling time between keys) were calculated in 39 MS patients. These were correlated against the Expanded Disability Status Scale (EDSS) scores, pyramidal and cerebellar functional system scores and 9-hole peg test scores.

**Results:**

EDSS correlated with KS (*r* = − 0.594, *p* < 0.001), AT (*r* = 0.464, *p* = 0.003) and IS (*r* = 0.423, *p* = 0.007). 9-HPT scores strongly correlated with KS (*r* = 0.926, *p* < 0.001). Pyramidal scores correlated with KS (*r* = − 0.517, *p* < 0.001). Cerebellar scores correlated with KS (*r* = − 0.665, *p* < 0.001), AT (*r* = 0.567, *p* < 0.001) and IS (*r* = 0.546, *p* = 0.007). Receiver operating characteristic curves demonstrate that KS can distinguish between the presence or absence of pyramidal and cerebellar dysfunction with area under curve 0.840 (*p* < 0.001) and 0.829 (*p* < 0.001), respectively.

**Conclusions:**

The BRAIN test can remotely measure disability in MS. Specific scores differ according to the presence and severity of pyramidal or extrapyramidal dysfunction. It demonstrates huge potential in monitoring disease progression in clinical trials.

**Electronic supplementary material:**

The online version of this article (10.1007/s00415-017-8690-x) contains supplementary material, which is available to authorized users.

## Introduction

A variety of outcome measures are available to assess upper limb function in multiple sclerosis (MS) [1]. The 9-hole peg test and handgrip strength are most frequently used in clinical trials and several technology-supported assessment tools such as the MS performance test (MPST) and virtual peg insertion test (VPIT) are now available [2–5]. All of these measures require direct administration by a clinician [1]. Remote monitoring of neurological disease activity has the potential to provide quantitative outcome measures with reduction of additional costs or inconvenience to patients [6]. Consumer-friendly accelerometers, such as FitBit, provide a promising means to remotely monitor ambulation in multiple sclerosis, however, there is an absence of tools for remote monitoring of upper limb function in MS [7].

The BRadykinesia Akinesia INcordination (BRAIN) test is a computer keyboard-tapping test that was developed to assess upper limb function in neurological disease. It has been validated as a sensitive tool for detecting signs of Parkinson’s disease and can be completed in the outpatient department, at home and in clinical trials using online software [8, 9].

The test involves alternately tapping keys at the opposite end of a keyboard using one finger. Subjects perform this as fast and accurately as possible during a 30 s period and this is then repeated with the other hand to derive several scores. The Kinesia Score (KS) measures the number of taps in 30 s, the akinesia time (AT) measures the mean dwell time on each key in milliseconds and the incoordination score (IS) measures the variance of the time interval between keystrokes in milliseconds.

In Parkinson’s disease, KS has been shown to be significantly lower than controls and is correlated to the severity of motor dysfunction, as measured by the UPDRS motor score (Spearman’s *r* = − 0.53, *p* < 0.001) [8]. In the same study, AT and IS were higher than in controls and weakly correlated with the degree of motor dysfunction (*r* = 0.27, *p* = 0.03 and *r* = 0.28, *p* = 0.03 respectively). A study using an earlier version of the BRAIN test in 12 patients with cerebellar dysfunction from a range of aetiologies found that KS was correlated to the score on the (unvalidated) Cerebellar Disease Rating Scale (*r* = − 0.38, *p* = 0.03) [10]. AT and IS did not correlate significantly with the degree of cerebellar dysfunction, but the study was limited by a small sample size (*r* = − 0.55, *p* = 0.06 and *r* = 0.30, *p* = 0.30, respectively).

Preliminary (unpublished) data from our group derived from a service development study using fampradine in MS patients showed that KS correlated strongly with EDSS (*r* = − 0.59, *p* < 0.001). The BRAIN test had not previously been studied in MS patients. In the present study, we aimed to confirm whether the BRAIN test could be used to measure disability in MS and to further characterise how KS, AT and IS vary in patients with pyramidal and cerebellar dysfunction.

## Methods

Patients with multiple sclerosis were recruited from the Royal London Hospital multiple sclerosis clinic. Demographic data for each subject was ascertained by interview and review of the medical records. This included gender, age, self-reported handed dominance and disease duration. Participants were then examined to determine the Expanded Disability Status Scale (EDSS) score by a trained clinician [11]. In determining this score, the eight functional system (FS) scores (pyramidal, cerebellar, brainstem, sensory, bowel and bladder, visual, cerebral and other) were recorded. The FS scores for pyramidal and cerebellar functions are shown in supplementary materials.

Participants undertook the BRAIN test following the EDSS assessment. They were seated at a desktop computer with a keyboard and followed on-screen test instructions from the BRAIN test website. This included being asked to alternately tap the ‘s’ and ‘;’ on the keyboard as fast and as accurately as possible during a 30 s period. They performed the test once using each hand and received no assistance during the test. Raw data from the BRAIN test website was used to determine the KS, AT and IS for each hand. The mean KS, mean AT and mean IS for each subject was then calculated. Subjects were also asked to perform the 9-hole peg test (9HPT). They were given standardised instructions and the scores were calculated, as previously described [1]. The 9HPT scores are, therefore, given as a reciprocal of the mean number of seconds to complete each trial.

Descriptive statistics for these variables were calculated. For continuous variables, means were reported if the data were normally distributed (assessed using the Shapiro–Wilks test) and medians were reported if not normally distributed. Associations between BRAIN test, EDSS, FS and 9HPT scores were estimated using Spearman’s rank correlation coefficient for non-normally distributed data. These correlation coefficients were compared using Steiger’s *Z* test with Fisher’s *r*-to-*z* transformation. Linear regression analysis was used to determine the relationship between BRAIN tests and EDSS scores. The *t* test and Mann–Whitney test were used to compare BRAIN test scores in those patients with or without pyramidal or cerebellar dysfunction and receiver operating characteristic (ROC) curves were used to compare the ability of BRAIN test scores to distinguish patients with or without any pyramidal or cerebellar dysfunction. The pre-determined significance level for all calculations was *p* = 0.01. All analyses were performed using GraphPad Prism version 6 for Mac.

The study was approved by the Queen Square Research Ethics Committee (09/H0716/48) and has, therefore, been performed in accordance with the ethical standards laid down in the 1964 Declaration of Helsinki and its later amendments. All participants provided written informed consent prior to inclusion in the study.

## Results

Assessments were performed on 39 patients with MS; three participants declined to undertake the 9-HPT and a further three did not complete the task within a minute and were assigned a value of 60 s. The mean age was 43.2 years (range 23–70), 74.4% of participants were female and 89.7% were right-handed.

The median disease duration was 4 years and the median EDSS score was 3 (range 0–6.5). The median pyramidal FS score was 2 (range 0–3) and median cerebellar FS score was 1 (range 0–4). The mean KS was 55.6 taps (range 21.5–87.5), the median AT was 96.5 ms (range 50.0–210.0) and the median IS was 10,780 ms^2^ (range 864–266,237). The median 9-HPT score was 0.047 s^−1^ (range 0.024–0.065).

**Fig. 1:**
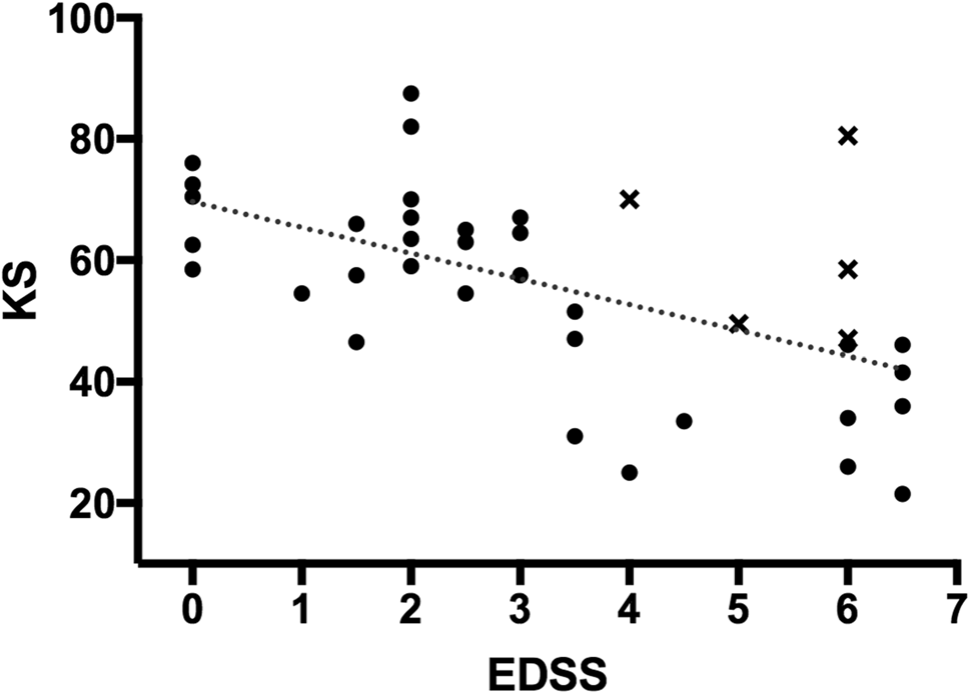
The distribution of KS scores according to EDSS. Data points marked with a cross indicate participants who had a paraparesis without upper limb pyramidal weakness

The EDSS correlated with KS, AT and IS. The correlation coefficients for KS (*r* = − 0.594, *p* < 0.001), AT (*r* = 0.464, *p* = 0.003) and IS (*r* = 0.423, *p* = 0.007) scores with EDSS were not significantly different from each other (*z* = 1.23, *p* = 0.211). The distribution of KS according to EDSS is shown in Fig. 1. Data points marked with a cross indicate those patients with a paraparesis in the absence of upper limb pyramidal weakness. An increase in the EDSS of 1 point was associated with 4.24 fewer taps over 30 s (95% CI 2.19–6.29).

The 9-HPT scores strongly correlated with KS (*r* = 0.926, *p* < 0.001). As expected, the 9-HPT scores correlated with EDSS (*r* = − 0.640, *p* < 0.001). There was no significant difference between the correlation coefficients for 9-HPT and KS with EDSS (*z* = − 0.439, *p* = 0.660).

**Fig. 2:**
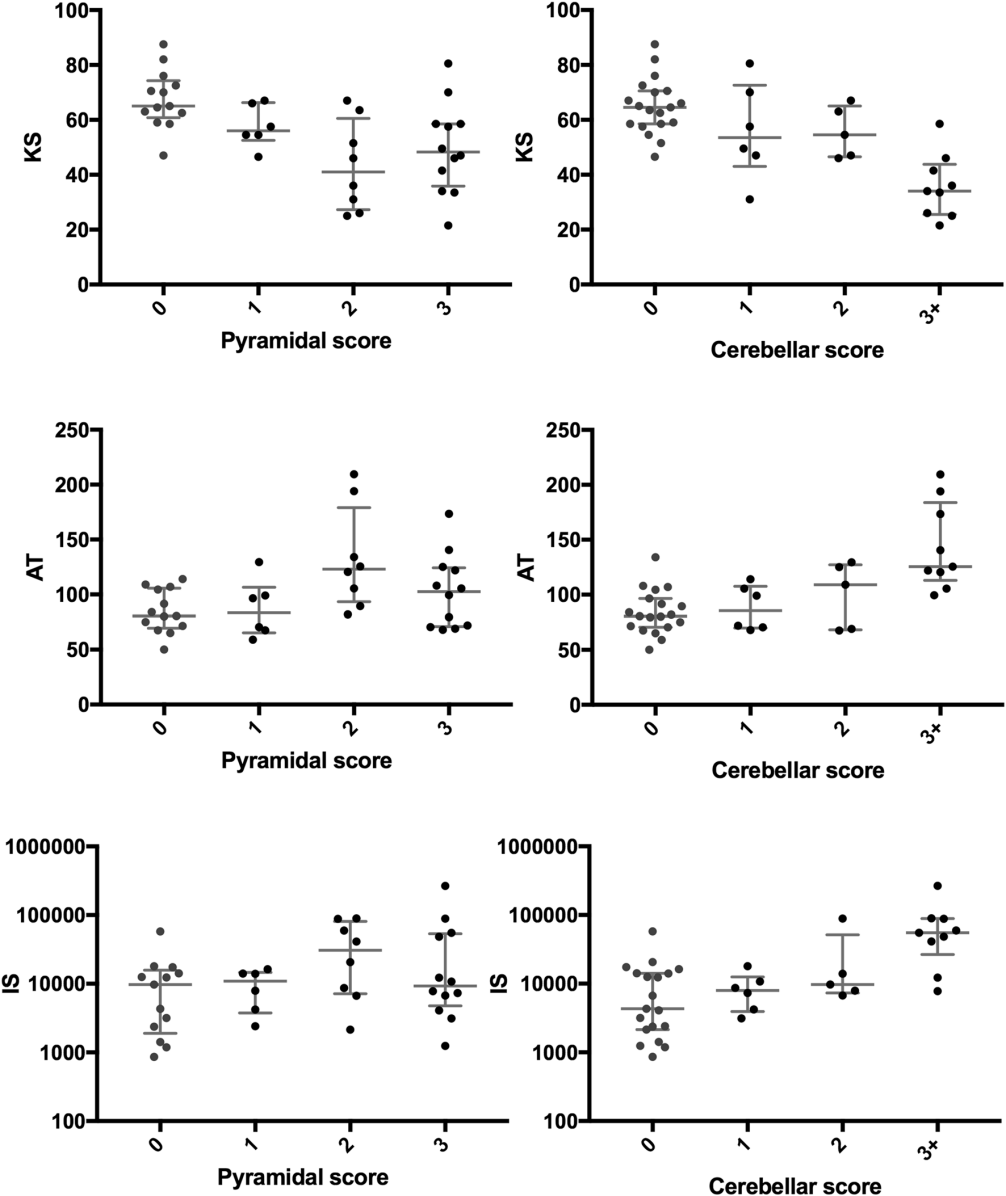
Each graph depicts the distribution of KS, AT or IS scores for each pyramidal or cerebellar score. The error bars show the median and interquartile ranges

Pyramidal dysfunction was associated with abnormal KS. The mean KS was lower in those patients with any pyramidal dysfunction compared to those without (49.6 vs 67.5 taps, *p* < 0.001) and KS correlated to the pyramidal score (*r* = − 0.517, *p* < 0.001). AT and IS were not significantly different in patients with pyramidal dysfunction and did not correlate with pyramidal score, as shown in Table 1. The distribution of these KS, AT and IS for varying degrees of pyramidal function is shown in Fig. 2.

**Table 1:** Spearman’s correlation coefficients and *p* values for KS, AT and IS with pyramidal and cerebellar scores

	Pyramidal score		Cerebellar score	
	*r*	*p* value	*r*	*p* value
KS	− 0.517	< 0.001	− 0.665	< 0.001
AT	0.279	0.085	0.567	< 0.001
IS	0.187	0.255	0.546	0.007

Cerebellar dysfunction was associated with abnormal KS, AT and IS. The mean KS was lower in those patients with cerebellar dysfunction compared to those without (46.8 vs 64.9 taps, *p* < 0.001) and KS correlated to the cerebellar score (*r* = − 0.665, *p* < 0.001). The median AT was higher in those patients with any cerebellar dysfunction compared to those without (112.0 vs 80.5 ms, *p* < 0.006) and AT correlated to the cerebellar score (*r* = 0.567, *p* < 0.001). The median IS was higher in those patients with any cerebellar dysfunction compared to those without (4317 vs 13,115 ms^2^, *p* < 0.006) and IS correlated to the cerebellar score (*r* = 0.546, *p* = 0.007). The Spearman’s correlation coefficients and *p* values for KS, AT and IS with pyramidal and cerebellar scores are shown in Table 1. Comparison between BRAIN test scores in patients with and without pyramidal or cerebellar dysfunction are summarized in the supplementary material. The distribution of KS, AT and IS for varying degrees of cerebellar function is shown in Fig. 2.

The BRAIN test distinguished between the presence or absence of pyramidal and cerebellar dysfunction. ROC curve analysis shows that KS can distinguish between the presence or absence of pyramidal dysfunction with an area under curve (AUC) of 0.840 (*p* < 0.001) and cerebellar dysfunction with an AUC of 0.829 (*p* < 0.001). AT and IS can distinguish between the presence or absence of cerebellar dysfunction with an area under curve (AUC) of 0.723 (*p* = 0.007) and 0.745 (*p* = 0.009), respectively, and as shown in Table 2. Most of the patients with cerebellar dysfunction also had significant pyramidal dysfunction (19 of 21 patients). The two patients with scores of ≥ 1 in the cerebellar domain and normal pyramidal scores had an average KS 55, AT 111.5 and IS 13,850, suggesting that AT and IS may be more specific for cerebellar dysfunction. Conversely, patients with scores of ≥ 1 in the pyramidal domain and normal cerebellar scores (8 of 27 patients) had an average KS 58, AT 89.9 and IS 8430. Patients with deficits in both domains had the most abnormal scores with average KS 45, AT 116 and IS 45,555, respectively.

**Table 2:** The area under curve (AUC) and *p* values for KS, AT and IS between MS patients with and without any pyramidal or cerebellar dysfunction

	Pyramidal dysfunction		Cerebellar dysfunction	
	AUC	*p* value	AUC	*p* value
KS	0.840	< 0.001	0.829	< 0.001
AT	0.673	0.081	0.723	0.007
IS	0.633	0.096	0.745	0.009

## Discussion

BRAIN test scores have previously been shown to differ between patients with Parkinson’s disease and controls and to correlate with Parkinson’s disease severity, as measured by the motor UPDRS [8, 9]. In the present study, we apply this clinical tool to patients with MS for the first time. We demonstrate that it can be used to remotely measure disease severity in multiple sclerosis and that specific scores differ according to the presence and severity of pyramidal or cerebellar dysfunction.

KS, AT and IS were highly correlated with EDSS scores in MS patients. KS appeared to be similar to the 9-HPT in measuring disability in MS and demonstrated particularly strong correlation with 9-HPT scores. Given that the BRAIN test can be administered online, this raises the possibility of using it in clinical trials for assessing disability in patients from their own home. In a previous study where controls were asked to repeat the test five times there was only a minimal learning effect with KS improving by 1.2 taps and this was not seen with AT and IS [8]. This highlights the potential for serial remote monitoring of disability in MS during trials, minimizing repeated time-consuming and costly hospital visits.

Analysis of BRAIN test scores in MS patients with or without pyramidal and cerebellar dysfunction, in conjunction with the correlations of BRAIN test scores to FS scores, demonstrated important differences between specific BRAIN test scores. Pyramidal dysfunction is associated with abnormal KS alone and the severity of pyramidal dysfunction correlates with KS. In contrast, cerebellar dysfunction was associated with abnormal KS, AT and IS and the severity of cerebellar dysfunction correlates with each of these scores. The caveat to these observations is that a large proportion of participants had both pyramidal and cerebellar dysfunction. In the aforementioned study, parkinsonism was associated with abnormal KS, AT and IS and the severity of parkinsonism (as measured by UPDRS) was correlated with KS (*r* = − 0.53, *p* < 0.001) and to a lesser degree AT (*r* = 0.27, *p* = 0.03) and IS (*r* = 0.28 and *p* = 0.03) [8].

KS, therefore, appears to be a non-specific marker of upper limb neurological impairment; reduced in parkinsonism, pyramidal and cerebellar dysfunction. This may be because it counts key taps over a given time period and in essence aggregates multiple facets of a complex motor task into a single and simple end point. By contrast, AT and IS measure subtle variations in a specific aspect of a repetitive movement. Use of the three scores in parallel may help to distinguish extrapyramidal from pyramidal dysfunction. Overall, these findings give new insights into the versatility of the BRAIN test. This quantitative approach to assessing upper limb function in MS also reinforces our understanding of the nature of specific movement disorders in that loss of rhythm generation and prolonged dwell times are seen in cerebellar dysfunction and are relatively preserved in pyramidal dysfunction.

An important limitation in using the BRAIN test to assess disability in MS, and other diseases that affect pyramidal or cerebellar function, is that it exclusively assesses upper limb function. EDSS scores are derived from the assessment of a combination of functional systems, disability in which may not affect upper limb function, and therefore, might not be detected by the BRAIN test. EDSS scores are also strongly influenced by ambulation. This limitation is highlighted in Fig. 1 that illustrates that patients with spastic paraparesis without upper limb pyramidal involvement have relatively preserved KS. Despite the inclusion of such patients in our study, KS remains highly correlated to pyramidal and EDSS scores. An additional limitation of this study is that we did not prospectively assess changes in BRAIN test scores with EDSS over time. The ability of the BRAIN test to detect changes in EDSS in a given patient over time needs to be addressed in a larger longitudinal study or clinical trial if this test can be used to monitor response to treatments.

In conclusion, this study has validated the BRAIN test as an online clinical tool that can remotely assess disability in MS and therefore demonstrates huge potential for monitoring disease progression in clinical trials. Our further understanding of KS, AT and IS will guide our interpretation BRAIN test scores in patients with parkinsonism, pyramidal and cerebellar dysfunction in the future and raises the possibility of an application for this test as an outcome measure in a broad spectrum of neurological diseases.

## Electronic supplementary material

Below is the link to the electronic supplementary material.

Supplementary material 1 (PDF 88 kb)
