# Supplementary material for: The BRAIN test: a keyboard-tapping test to assess disability and clinical features of multiple sclerosis
Source: J Neurol. 2017 Dec 4;265(2):285–90. doi: 10.1007/s00415-017-8690-x (PMC5808056; doi:10.1007/s00415-017-8690-x)
Supplement: Supplementary file 1 — Supplementary material 1 (PDF 88 kb) [file 415_2017_8690_MOESM1_ESM.pdf]

Pyramidal Function	
**0**	Normal
**1**	Abnormal Signs without disability
**2**	Minimal disability
**3**	Mild-to-moderate hemiparesis/paraparesis *or* severe monoparesis
**4**	Marked paraparesis/hemiparesis *or* moderate tetraparesis
**5**	Paraplegia/hemipelgia *or* marked tetraparesis
**6**	Tetraplegia

Table of Functional System (FS) score for pyramidal function (Kutzke et al, 1983)

Cerebellar Function	
**0**	Normal
**1**	Abnormal Signs without disability
**2**	Mild ataxia *or* moderate station ataxia *or* tandem gait not possible
**3**	Moderate limb ataxia *or* moderate gait/truncal ataxia
**4**	Severe gait/truncal ataxia *and* severe ataxia in at least 3 limbs
**5**	Unable to perform co-ordinated movements due to ataxia
**X**	Pyramidal weakness interfere with cerebellar testing

Table of Functional System (FS) score for cerebellar function (Kutzke et al, 1983)

	**Pyramidal dysfunction**			**Cerebellar dysfunction**		
	**Absent**	**Present**	**p-value**	**Absent**	**Present**	**p-value**
**KS** (taps)	67.5	49.6	<0.001	64.9	46.8	<0.001
**AT** (msec)	80.5	103.0	0.083	80.5	112.0	0.006
**IS** ($msec^2$)	9742	11509	0.187	4317	13115	0.008

Table of comparison between BRAIN test scores in patients with and without pyramidal or cerebellar dysfunction. Mean scores with p-values according to the unpaired t-test are shown for KS and median scores with p-values according to the Mann Whitney test are shown for AT and IS.
